# Functional Analysis of Rare Genetic Variants in the Negative Regulator of Intracellular Calcium Signaling RCAS/SLC10A7

**DOI:** 10.3389/fmolb.2021.741946

**Published:** 2021-10-04

**Authors:** Marie Wannowius, Emre Karakus, Joachim Geyer

**Affiliations:** Institute of Pharmacology and Toxicology, Faculty of Veterinary Medicine, Justus Liebig University Giessen, Giessen, Germany

**Keywords:** SLC10A7, RCAS, calcium signaling, STIM, co-localization, rare genetic variant

## Abstract

The solute carrier family 10 member SLC10A7 is a negative regulator of intracellular calcium signaling (RCAS). In cell culture, SLC10A7 expression is negatively correlated with store-operated calcium entry (SOCE) via the plasma membrane. SLC10A7-deficient cells have significantly increased calcium influx after treatment with thapsigargin for depletion of ER calcium stores, whereas SLC10A7/RCAS overexpression limits calcium influx. Genetic variants in the human *SLC10A7* gene are associated with skeletal dysplasia and amelogenesis imperfecta and reveal loss of function on cellular calcium influx. More recently, an additional disease-related genetic variant (P303L) as well as some novel genetic variants (V235F, T221M, I136M, L210F, P285L, and G146S) have been identified. In the present study, these variants were expressed in HEK293 cells to study their subcellular localization and their effect on cellular calcium influx. All variants were properly sorted to the ER compartment and closely co-localized with the STIM protein, a functional component of SOCE. The variants P303L and L210F showed significantly reduced effects on cellular calcium influx compared to the wild type but still maintained some degree of residual activity. This might explain the milder phenotype of patients bearing the P303L variant and might indicate disease potential for the newly identified L210F variant. In contrast, all other variants behaved like the wild type. In conclusion, the occurrence of variants in the *SLC10A7* gene should be considered in patients with skeletal dysplasia and amelogenesis imperfecta. In addition to the already established variants, the present study identifies another potential disease-related SLC10A7/RCAS variant, namely, L210F, which seems to be most frequent in South Asian populations.

## Introduction

Calcium (Ca^2+^) is one of the most important regulatory ions of eukaryotic cells and is involved in many physiological and cellular processes. The concentration of free Ca^2+^ ions is much higher in the extracellular than in the intracellular compartment. In addition, Ca^2+^ is sequestered in the endoplasmic and sarcoplasmic reticulum (ER/SR), from where it can be released for rapid cellular signaling (Fahrner et al., 2018). In response to ER calcium depletion, store-operated calcium entry (SOCE) is activated and allows Ca^2+^ to enter cells via the plasma membrane (Fahrner et al., 2018). The stromal interaction molecule STIM and ORAI (named for the keeper of the gates to heaven in Greek mythology; Feske et al., 2006) are the major functional components of SOCE. STIM, with its Ca^2+^ binding domain (EF hand) in the luminal side of the ER, represents a Ca^2+^ sensor (Liou et al., 2005; Roos et al., 2005; Stathopulos et al., 2008). When the concentration of Ca^2+^ in the ER is decreased by IP_3_-induced Ca^2+^ release, Ca^2+^ dissociates from the EF hand of the STIM molecule (Woo et al., 2018). Subsequently, STIM is translocated to so-called ER-PM junctions near the plasma membrane, where it interacts with ORAI (Fahrner et al., 2018). ORAI is localized in the plasma membrane and after interacting with STIM allows Ca^2+^ influx into the cell (Stathopulos et al., 2008). The ER/SR Ca^2+^-ATPase SERCA, which is specifically located in the ER membrane, is then responsible for refilling the ER Ca^2+^ stores (Krebs et al., 2015). Once the Ca^2+^ stores of the ER are refilled, STIM dissociates from ORAI, and Ca^2+^ influx into the cell via this calcium release activated channel (CRAC) is terminated (Fahrner et al., 2018).

Recently, we identified a novel negative regulator of intracellular Ca^2+^ signaling, namely RCAS. RCAS (gene symbol: *SLC10A7*) belongs to the solute carrier family 10 of bile acid and steroid sulfate membrane transporters (Karakus et al., 2020). In cell culture, SLC10A7/RCAS expression was negatively correlated with calcium influx via the plasma membrane (Karakus et al., 2020). SLC10A7-deficient cells had significantly increased Ca^2+^ influx after treatment with thapsigargin (TG), ionomycin, and ATP/carbachol treatment, which are commonly used for depletion of ER Ca^2+^ stores. Furthermore, SLC10A7-deficient cells showed significantly higher intracellular Ca^2+^ levels. In contrast, SLC10A7/RCAS overexpression significantly reduced the Ca^2+^ influx, clearly pointing to a role of the SLC10A7/RCAS protein as negative regulator of intracellular calcium signaling. However, the exact molecular function of SLC10A7/RCAS as a transporter or regulator molecule is still not clear. However, there are several hypotheses about its function. (I) SLC10A7 might limit the transport capacity of SERCA or might increase the rate of Ca^2+^ leaking from the ER. (II) SLC10A7 might negatively regulate STIM and/or ORAI, e.g., by affecting the sensitivity of STIM to Ca^2+^, or by decreasing the probability of ORAI opening. (III) SLC10A7/RCAS might also play a role for STIM-ORAI complex formation or the stability of this complex at the plasma membrane (Karakus et al., 2020).

In three independent studies, mutations in the human *SLC10A7* gene were associated with a severe disease phenotype characterized by skeletal dysplasia with short stature, osteoporosis, amelogenesis imperfecta, skeletal deformations, facial abnormalities, visual and hearing impairment, and intellectual disability (Ashikov et al., 2018; Dubail et al., 2018; Laugel-Haushalter et al., 2019). Supporting this observation, in one study *Slc10a7* knockout mice showed similar phenotypic characteristics as human patients with SLC10A7 mutations, including abnormal skeletal development and dental anomalies (Dubail et al., 2018). In addition, studies in zebrafish with *slc10a7* morpholino knockdown have shown defective bone mineralization, which leads to the hypothesis that SLC10A7/RCAS plays an essential role in cartilage formation and bone development (Ashikov et al., 2018). Moreover, biochemical analyses of patients with *SLC10A7* gene mutations have revealed abnormal N-glycosylation of the plasma protein transferrin as well as mislocalization and defective post-Golgi transport of glycoproteins (Ashikov et al., 2018). Although not proven yet, these effects might be due to dysregulation of the Ca^2+^ homeostasis under *SLC10A7* mutation.

Several *SLC10A7* mutations previously described to be associated with human pathologies have been functionally analyzed for their effect on Ca^2+^ signaling after treatment with TG in cell culture (Karakus et al., 2020). These include the splice-site mutations c.774-1G > A (leading to the skipping of exons 9 and 10 or only exon 10) and c.773 + 1G > A and c.722-16A > G (both leading to the skipping of exon 9) as well as the missense mutations c.388G > A (G130R), c.221T > C (L74P), and c.335G > A (G112D) (Ashikov et al., 2018; Dubail et al., 2018; Laugel-Haushalter et al., 2019). Compared to the wild-type SLC10A7 construct, none of these mutants had a significant effect on Ca^2+^ signaling, thus indicating a loss-of-function phenotype (Karakus et al., 2020). The exception is the mutant G112D, which has a moderate but significant residual function. In contrast, the missense mutation P303L described more recently (Laugel-Haushalter et al., 2019) has not been functionally analyzed so far. In addition, several rare missense genetic variants with different rates of occurrence in specific ethnic groups have been identified in the *SLC10A7* gene. These variants could also affect the function of the RCAS protein based on bioinformatics analyses.

Therefore, the aim of the present study was to functionally characterize these missense *SLC10A7* genetic variants by measuring SOCE in HEK293 cells transfected with the respective variants. Among six novel genetic *SLC10A7* variants, the present study identified the variant L210F that is most frequent in South Asian populations as a novel potential disease-related SLC10A7/RCAS variant.

## Material and Methods

### Materials

Unless otherwise stated, all chemicals, including TG (T9033) and probenecid (P8761), were from Sigma-Aldrich (Taufkirchen, Germany). Fluo-4 AM (F14201) was purchased from Thermo Scientific (Waltham, MA, United States). Ca^2+^-free HEPES buffer was prepared as follows: NaCl 140 mM, KCl 4 mM, HEPES 10 mM, MgCl_2_ 1 mM, and glucose 25 mM (pH 7.4).

### Cell Culture

GripTite 293 MSR cells (hereafter, “HEK293 cells”) were maintained in Dulbecco’s Modified Eagle Medium (Gibco, Carlsbad, CA, United States) supplemented with 10% fetal calf serum (Pan-Biotech, Aidenbach, Germany), 1% Minimum Essential Medium of Non Essential Amino Acids (Gibco), L-glutamine (4 mM; Anprotec, Bruckberg, Germany), penicillin (100 U/ mL; Anprotec), and streptomycin (100 μg/ ml; Anprotec) at 37°C, 5% CO_2_, and 95% humidity.

### Generation of Fluorescence Constructs

C-terminally mScarlet-tagged constructs for STIM1, ORAI1, and SERCA2b were generated as reported previously for SLC10A7 transcription variant v2 (hereafter, “SLC10A7/RCAS wild-type (WT)”; Noppes et al., 2019). Flexible linker protein sequences (see Table 1) followed by the cDNA sequence coding for the monomeric red fluorescent protein mScarlet were added virtually to the constructs via DNASTAR 16.0 SeqBuilder Pro and were synthesized by Biocat (Heidelberg, Germany) into the pcDNA3.1 (+) expression vector. The C-terminally GFP-tagged STIM1 transcript variant 2 plasmid was generated by amplifying the STIM1 sequence out of HEK293 cDNA using Phusion Flash PCR Master Mix (F-548; Thermo Scientific) with the following primers: 5′-acc atg gat gta tgc gtc cgt ctt gcc ctg t-3′ forward and 5′-ctt ctt aag agg ctt ctt aaa gat ttt gag ggg aaa ctt ctt ccg-3′ reverse. PCR amplification was performed on a peqSTAR XS PEQLAB PCR cycler for 27 cycles under the following conditions: initialization for 10 s at 98°C, denaturation for 1 s at 98°C, annealing for 15 s at 62°C, extension for 150 s at 72°C, final elongation for 1 min at 72°C, and a final hold at 4°C. After amplification, the PCR product was separated by 1% agarose gel electrophoresis and the appropriate band was excised and purified with a GeneJET PCR Purification Kit (#K0702; Thermo Scientific). Subsequently, the product was A-tailed with dATP nucleotides and Taq DNA Polymerase (#EP0402; Thermo Scientific) for 150 min at 72°C. Then the product was cloned into pcDNA6.2/C-EmGFP-Gw/TOPO Cloning Vector (Thermo Scientific) and immediately transformed into TOP10 chemically competent *Escherichia coli* (*E. coli*). Grown colonies were picked and plasmids were isolated with the GeneJET Plasmid Miniprep Kit (Thermo Scientific) according to the manufacturer’s protocol. Finally, the generated STIM-GFP was verified by DNA sequencing (Seqlab Microsynth).

**TABLE 1:** Flexible linker amino acid sequences inserted between the corresponding protein and the mScarlet fluorescence tag in the pcDNA3.1 (+) vector.

Protein	Flexible linker amino acid sequence
SLC10A7	GGGGSGGGGSGGGG
STIM1	SGGGGSGGGGSGGGGS
ORAI1	SGGGGSGGGGSGGGGS
SERCA2b	SGGGGSGGGGSGGGGS

### Site-Directed Mutagenesis

Site-directed mutagenesis was performed to create the described point mutations in SLC10A7-v2-mScarlet (in pcDNA3.1 (+) vector). Forward and reverse primers were designed and are listed in Table 2. Amplification was performed on a peqSTAR XS PEQLAB PCR cycler with Pfu DNA Polymerase (M774A; Promega, Madison, WI, United States) at 18 cycles under the following conditions: initial denaturation for 2 min at 95°C, denaturation for 30 s at 95°C, annealing for 1 min at 55°C, extension for 8.5 min at 72°C, final elongation for 10 min at 72°C, and a final hold at 4°C. After amplification, products were digested with *Dpn*I enzyme (ER1701; Thermo Scientific) for 1 h at 37°C and transformed into TOP10 chemically competent *E. coli*. Plasmids were isolated with a GeneJET Plasmid Miniprep Kit (Thermo Scientific) according to the manufacturer’s protocol, and the generated point mutations were verified by DNA sequencing (Seqlab Microsynth).

**TABLE 2:** Primers used for site-directed mutagenesis of the SLC10A7-v2-mScarlet construct. *, stop codon.

Amino acid substitution	Nucleotide substitution	Primer sequences (5′ → 3′)			
		Forward	Tm (°C)	Reverse	Tm (°C)
V235F	GTT → TTT	aat​tca​gcc​ttt​ttc​tca​tac​tgt​t	54	tat​cca​ggt​caa​tat​ttg​ggt​tag​a	55
T221M	ACG → ATG	cat​tct​gtg​aca​tgt​tct​cta​acc​c	57	ttg​tgt​aga​tga​tca​tga​gga​gta​c	55
I136M	ATA → ATG	ggc​agc​tgc​aat​gtt​taa​ttc​agc​c	62	tca​ttt​cca​cca​act​gcc​ttg​gtt​a	61
L210F	CTC → TTC	agc​agc​agt​gta​ttc​ctc​atg​atc​a	60	gat​agc​acc​aaa​agg​agg​ctt​ctt​t	59
P285L	CCG → CTG	cct​tac​att​ggg​aat​tct​gat​gct​gaa​gat​cgt​gtt​tg	73	caa​aca​cga​tct​tca​gca​tca​gaa​ttc​cca​atg​taa​gg	73
G146S	GGC → AGC	gga​agt​ttt​ttg​agc​atc​gtt​ata​a	53	aaa​ggc​tga​att​aaa​tat​tgc​agc​t	55
P303L	CCC → CTC	taa​tat​ctg​tac​tct​tgc​tca​tct​acc	55	aag​aga​gat​gct​cat​ggc​ct	58
Q172*	CAG → TAG	cat​cta​ttt​ttt​ctt​agc​ttt​tta​tga​ctg	52	tga​aag​gca​cag​aag​aag​atg	54
L74P	CTT → CCT	ggt​gca​tct​aaa​act​gca​tcc​ttt​tat​tca​gat​ctt​tac​tct​tgc​att​ctt​ccc​ag	76	ctg​gga​aga​atg​caa​gag​taa​aga​tct​gaa​taa​aag​gat​gca​gtt​tta​gat​gca​cc	76

### Calcium Imaging

For Ca^2+^ imaging, HEK293 cells (6.0 × 10^4^ per well) were seeded into 96-well plates (83.3924; Sarstedt, Nümbrecht, Germany) coated with poly-L-lysine. Then 6 h after seeding, the cells were transiently transfected with 0.5 µg SLC10A7-mScarlet WT or mutant plasmid DNA with Lipofectamine 2000 (11668–019; Invitrogen, Carlsbad, CA, United States) according to the manufacturer’s protocol. After 40 h of culturing, the medium was replaced with fresh serum-free medium for 2 h. Subsequently, the cells were incubated in Ca^2+^-free HEPES buffer containing 2 µM Fluo-4 AM and 1 mM probenecid for 15 min at room temperature. Afterward, the cells were washed gently three times with Ca^2+^-free HEPES buffer and incubated for an additional 15 min to allow complete de-esterification of intracellular AM esters. Then the cells were incubated with 1 μM TG at room temperature. After 5 min of treatment with TG basal fluorescence was recorded for 1 min with a DM5500 Leica fluorescent microscope (200-fold magnification, green [488 nm] and red [568/594 nm] filter sets; Leica, Wetzlar, Germany). After these 60 s, 2 mM Ca^2+^ (5239.2; Roth, Karlsruhe, Germany) was added and Ca^2+^-induced fluorescence was recorded every 10 s for an additional minute. The fluorescence signal was determined with LAS-X (Leica Application Suite X; Leica) for approximately 80 defined regions of interest of single cells. Data are presented as the mean background-subtracted fluorescence intensity of each cell normalized to the intensity of the first image (F/F0).

### Fluorescence Microscopy

For co-localization studies, HEK293 cells (1.0 × 10^4^ per well) were seeded into 8-well µ-slides (80826; IBIDI, Gräfelfing, Germany) coated with poly-L-lysine. Then 6 h after seeding, the cells were transiently transfected with 1 µg plasmid DNA (0.5 µg GFP construct and 0.5 µg mScarlet construct) with Lipofectamine 2000 (11668-019; Invitrogen) according to the manufacturer’s protocol. After 40 h of culturing, the cells were washed with PBS and incubated with or without 2 μM TG for 10 min at 37°C. Subsequently, the cells were washed with PBS three times and fixed with 3% PFA (0335.1; Roth) for 20 min at room temperature. Thereafter, the cells were washed and loaded with 200 µL PBS per well. Z-stack cell imaging of approximately 50 single cells per well was performed as described below. For co-localization with green fluorescent organelle markers, cells were seeded and transiently transfected as described above, except only 0.5 µg mScarlet construct (SLC10A7-/STIM1-/ORAI1-/SERCA2b-mScarlet) were used for Lipofectamine 2000 transfection. After 24 h of culturing, the cell medium was changed and approximately 30 particles per cell CellLight Reagent BacMam 2.0 (Invitrogen) containing a signal peptide fused to emerald GFP were added to allow expression in the following compartments: ER (C10590), late endosomes (C10588), lysosomes (C10596), early endosomes (C10586), Golgi (C10592), peroxisomes (C10604), and mitochondria (C10600). After 16 h of culturing, the cells were washed with PBS and fixed as described above. Then Z-stack cell imaging of approximately 5–48 single cells per well was performed. All co-localization studies were performed at room temperature on an inverted Leica DM5500 fluorescence microscope (Leica). Images were generated using a 63 × oil objective and green (488 nm) and red (568/594 nm) filter sets, respectively. The Z-stack (9.5 µm size) of single cells was recorded. Subsequently, Pearson’s correlation coefficients (PCC) were calculated with the LAS-X imaging software.

### Statistical Analysis

Statistical analysis was performed with the Student’s t-test and one-way ANOVA in GraphPad Prism 6.0 (GraphPad Software, San Diego, CA, United States). Error bars represent means ± SD. The number of samples, number of experimental repetitions, and significance level are indicated in the figure legends.

## Results

**FIGURE 1:**
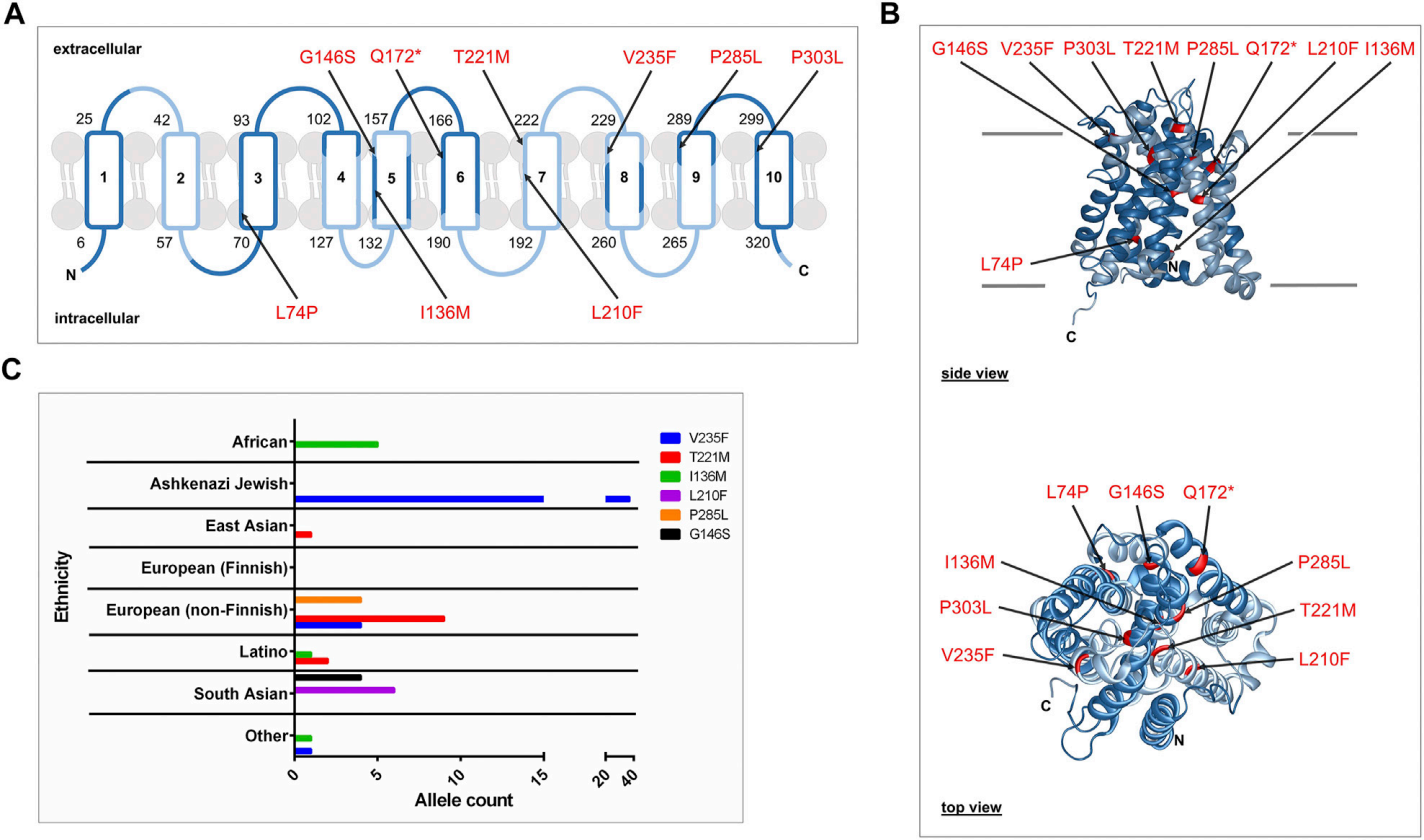
SLC10A7 rare genetic variants predicted to be damaging or possibly damaging. **(A)** Schematic membrane topology model of the human SLC10A7/RCAS protein isoform b with its 10 transmembrane domains (TMDs). This protein is coded by 12 exons, as indicated by alternating dark blue (exons 1, 3, 5, 7, 9, 11) and light blue (exons 2, 4, 6, 8, 10, 12) labeling. The N- and C-terminal ends are both located inside the cell. Numbers indicate the amino acid positions at the beginning and end of each TMD. Arrows show the localization of the nine SLC10A7 variants investigated here. **(B)** Side and top views of a 3D homology model of the human SLC10A7/RCAS protein. Variants are highlighted in red. The protein sequence with GenBank Accession No. NP_001025169 was used as a target sequence for SWISS-MODEL homology modeling (https://swissmodel.expasy.org). The homology model is based on the crystal structure of ASBT from *Yersinia frederiksenii* (PDB: 4n7w; Zhou et al., 2014) **(C)** Graphical representation of the variant allele counts in different ethnicities.

The Genome Aggregation Database (gnomAD v2.1.1, https://gnomad.broadinstitute.org) lists more than 650 genetic variants for the human *SLC10A7* gene. From these we filtered out the 540 variants that occur in exon sequences and further selected 140 missense variants (Supplementary Table S1). To improve the quality of the data and to avoid taking data from sequencing errors, we kept only those variants with an allele count >3. Then, we cut off the number of alleles at 50 to exclude relatively frequent variants. Of the 29 remaining variants, only six were predicted to affect protein function based on SIFT (https://sift.bii.a-star.edu.sg) and PolyPhen (https://genetics.bwh.harvard.edu) predictions, and these variants (V235F, T221M, I136M, L210F, P285L, and G146S) were experimentally analyzed in the present study (Table 3). The variants were distributed over the whole SLC10A7/RCAS protein, as indicated in 2D and 3D models (Figures 1A,B). They had overall allele counts of 4–42, and minor allele frequencies ranged from 0.0016% (G146S) to 0.0168% (V235F) (Table 4). It is interesting that all variants had different occurrences in specific ethnic groups. Most pronounced in this regard were the dominant allele counts of the V235F variant among Ashkenazi Jews (Figure 1C). In addition, three variants (Q172*, P303L, and L74P) were included that were previously associated with a disease phenotype (Dubail et al., 2018; Laugel-Haushalter et al., 2019). Data on minor allele frequency are not available for these variants.

**TABLE 3:** GnomAD selection algorithm for the selection of *SLC10A7* rare genetic variants.

Selection steps for *SLC10A7* rare genetic variants in the gnomAD v2.1.1 online tool	Hit number
Total number of gnomAD v2.1.1 variants for *SLC10A7*	658
Variants in coding exons	540
Variants in coding exons that were missense variants	140
Variants in coding exons that were missense variants and had an allele count from >3 but <50	29
Variants in coding exons that were missense variants and had an allele count from >3 but <50 and were predicted to affect protein function by SIFT analysis tool	7
Variants in coding exons that were missense variants and had an allele count from >3 but <50 and were predicted to affect protein function by SIFT analysis tool and by Polyphen prediction software.	6 (V235F, T221M, I136M, L210F, P285L, G146S)

**TABLE 4:** Overview of the analyzed SLC10A7 variants and their predicted effects on protein function. Minor allele frequencies are indicated. The last three variants listed were described in earlier studies (Dubail et al., 2018; Laugel-Haushalter et al., 2019). L74P served as a control in the present study, and P303L has not yet been functionally characterized.

SNP	Nucleotide position	Nucleotide substitution	Amino acid substitution	PolyPhen prediction	SIFT prediction	Allele count	Minor allele frequency (%)
rs148698801	703	GTT → TTT	V235F	possibly damaging	affected protein function	42	0.0168
rs201501147	662	ACG → ATG	T221M	probably damaging	affected protein function	12	0.0048
rs145454109	408	ATA → ATG	I136M	probably damaging	affected protein function	7	0.0031
rs764016906	628	CTC → TTC	L210F	probably damaging	affected protein function	6	0.0024
rs775994807	854	CCG → CTG	P285L	probably damaging	affected protein function	4	0.0017
rs758419384	436	GGC → AGC	G146S	probably damaging	affected protein function	4	0.0016
**Patient variant described by**							
−	908	CCC → CTC	P303L	probably damaging	affected protein function	Laugel-Haushalter et al. (2019)		
rs1376082145	514	CAG → TAG	Q172*	−	−	Dubail et al. (2018)		
rs1560980659	221	CTT → CCT	L74P	probably damaging	affected protein function	Dubail et al. (2018)		

**FIGURE 2:**
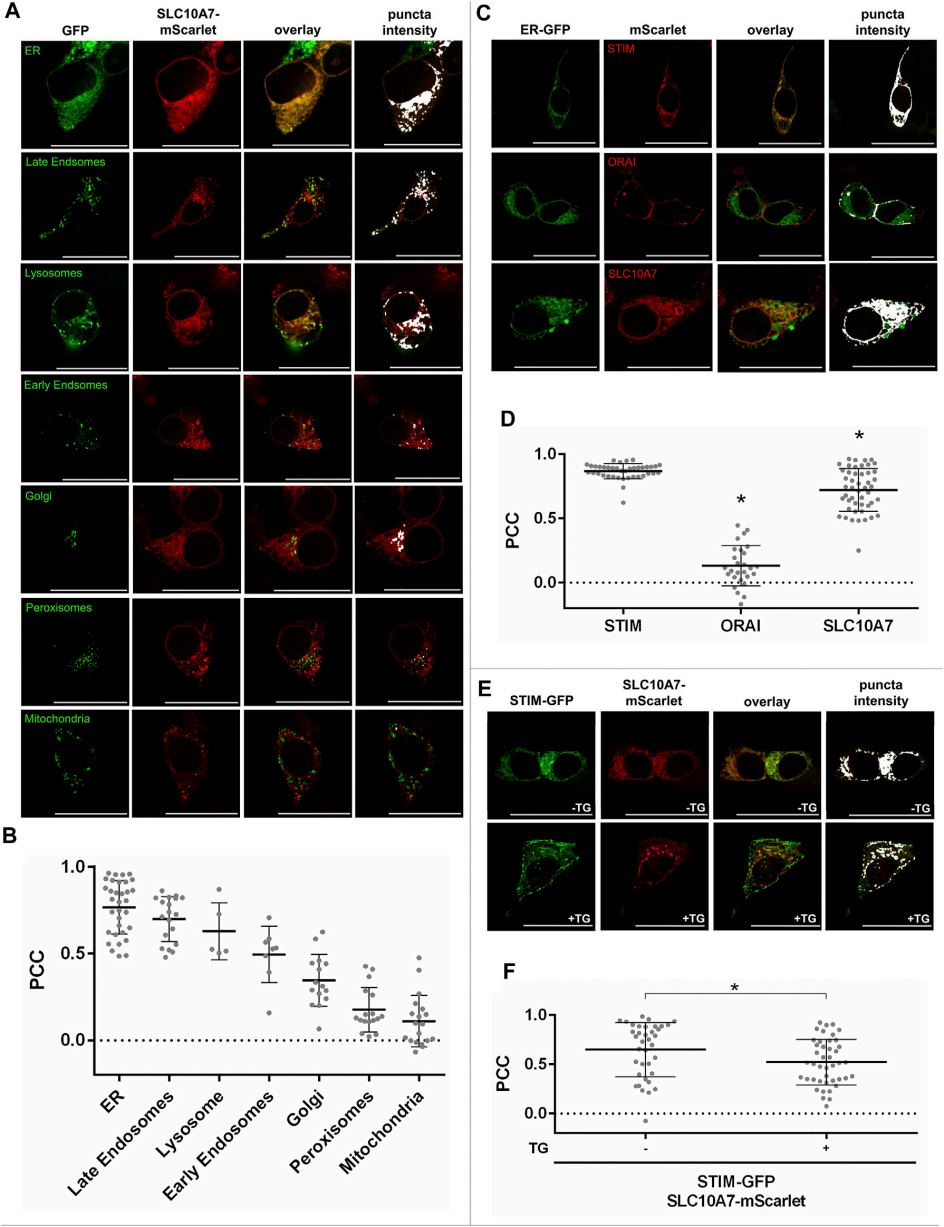
Cellular localization of the SLC10A7/RCAS protein and its co-localization with STIM and ORAI. **(A)** Co-localization of the SLC10A7-mScarlet construct (red fluorescence) with the indicated organelle markers (green fluorescence). Images represent maximum projections of Z-stacks at 630 × magnification after deconvolution. **(B)** Graphical representation of the co-fluorescence between SLC10A7-mScarlet and ER (*n* = 33), late endosomes (*n* = 19), lysosomes (*n* = 5), early endosomes (*n* = 8), Golgi (*n* = 15), peroxisomes (*n* = 17), and mitochondria (*n* = 19), expressed as Pearson’s correlation coefficient (PCC). Each dot represents the PCC of a single cell. Numbers in brackets indicate the number of cells analyzed. **(C)** Co-localization of the mScarlet-tagged STIM, ORAI, and SLC10A7 constructs with the GFP-tagged ER organelle marker. Images represent maximum projections of Z-stacks at 630 × magnification after deconvolution **(D)** Graphical representation of the co-fluorescence between the green fluorescent ER marker and the mScarlet-tagged STIM, ORAI, and SLC10A7/RCAS proteins, respectively, expressed as PCC. Each dot represents the PCC of a single cell. In total, 39 cells were analyzed for STIM, 27 for ORAI and 48 for SLC10A7. **(E)** Representative fluorescence images showing STIM-GFP/SLC10A7-mScarlet co-localization before **(upper pictures)** and after **(lower pictures)** treatment with thapsigargin (TG). Images represent maximum projections of Z-stacks at 630 × magnification after deconvolution. **(F)** Graphical representation of the co-fluorescence between STIM-GFP and SLC10A7-mScarlet, expressed as PCC before (–TG) and after (+TG) treatment with TG. Each dot represents the PCC of a single cell. In total, 37 (−TG) and 43 (+TG) single cells were analyzed. Data means ± SD are indicated with lines for a representative experiment. * Significantly different from all other groups at *p* < 0.05. Scale bars: 25 µm.

To functionally characterize these variants, we measured Ca^2+^ influx in transfected HEK293 cells as done before for the disease-related L74P, G112D, G130R, exon Δ9, exon Δ10, and exon Δ9 + 10 variants (Karakus et al., 2020). As variations in amino acids may change the sorting and/or functional properties of a protein, both processes were analyzed. We first examined whether the SLC10A7/RCAS variant proteins were sorted in the same way as the WT. Therefore, red fluorescent SLC10A7-mScarlet-tagged constructs were generated and transfected into HEK293 cells. In addition, different cell organelles were labeled with respective GFP-tagged marker proteins. As indicated in Figure 2A, the SLC10A7/RCAS protein mostly localized to the ER compartment, as indicated by the very high degree of fluorescence overlay. In addition, co-localization of the SLC10A7-mScarlet protein was detected with the organelle markers for late endosomes, lysosomes, early endosomes, and the Golgi compartment, whereas SLC10A7 showed hardly any signals in peroxisomes and mitochondria (Figure 2A). However, it has to be emphasized that for some organelle markers the expression rates were quite low and so only small numbers of cells could be analyzed for co-fluorescence. To estimate the degree of co-fluorescence, we calculated PCC (Figure 2B), which revealed the highest values for the ER compartment. In addition to SLC10A7-mScarlet, mScarlet-tagged constructs were generated for STIM, ORAI, and SERCA. It is interesting that, STIM-mScarlet (Figures 2C,D) and SERCA-mScarlet (Supplementary Figures S1A,B) showed an even higher degree of co-localization with the green fluorescent ER marker, with PCC values at about 0.86. In contrast, ORAI appeared weakly in the ER but was clearly detected in the plasma membrane, as expected (Figures 2C,D). Next co-localization of the SLC10A7-mScarlet construct with STIM-GFP was analyzed and revealed quite high PCC values of 0.65 that were significantly reduced after the cells were treated with TG to deplete Ca^2+^ stores in the ER (Figures 2E,F). This can be explained by the well-known translocation of STIM after TG treatment. In parallel, co-localization of STIM-GFP and SERCA-mScarlet decreased significantly after treatment with TG, but PCC values for STIM-GFP and ORAI-mScarlet co-localization increased, nicely reflecting the translocation of STIM toward the plasma membrane to form the STIM-ORAI complex that ultimately supports SOCE (Supplementary Figures S2A,B). To analyze whether the relatively large mScarlet tag would affect the sorting of the SLC10A7/RCAS protein, we used the small FLAG epitope as an additional tag to localize the protein. Co-expression of SLC10A7-mScarlet with the SLC10A7-FLAG construct revealed a very high degree of overlay, indicating identical sorting of these two proteins (data not shown).

**FIGURE 3:**
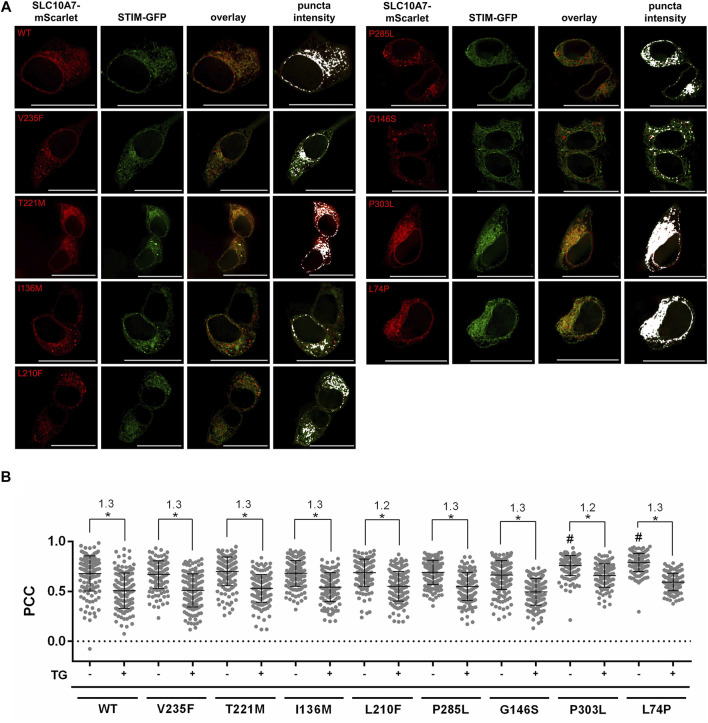
Co-localization of STIM-GFP with SLC10A7-mScarlet wild-type (WT) and mutant (V235F, T221M, I136M, L210F, P285L, G146S, P303L, and L74P) constructs before and after treatment with thapsigargin (TG). **(A)** Representative fluorescence images show GFP/mScarlet co-fluorescence before treatment with TG. Images represent maximum projections of Z-stacks at 630 × magnification after deconvolution. **(B)** Graphical representation of the co-fluorescence, expressed as Pearson’s correlation coefficient (PCC) before (–TG) and after (+TG) treatment with TG. Each dot represents the PCC of a single cell. Approximately 120–200 single cells per construct were analyzed. Ratios of the mean values of treated and untreated cells are also given. Means ± SD of three combined independent experiments (*n* = 3) of the measured PCCs are indicated by lines. * Significantly different at *p* < 0.05. # Significantly different from WT SLC10A7. Scale bars: 25 µm.

Based on these preliminary experiments, the sorting and localization of the SLC10A7 variants were analyzed compared to the WT SLC10A7/RCAS protein. As a marker for proper sorting and intact response to treatment with TG, we used the co-localization of the respective SLC10A7-mScarlet construct with the STIM-GFP construct in the presence and absence of TG (Figure 3A). Whereas the SLC10A7 variants V235F, T221M, I136M, L210F, P285L, and G146S showed degrees of co-localization with STIM comparable to those of the WT SLC10A7/RCAS protein, the disease-related variants P303L and L74P had significantly higher PCC values for co-localization with STIM-GFP. However, after treatment with TG, all variants decreased equally in their co-localization with STIM, with ratios of 1.2–1.3 for all constructs (Figure 3B). Note that the Q172* variant was not properly expressed in HEK293 cells, and therefore this variant could not be analyzed further.

**FIGURE 4:**
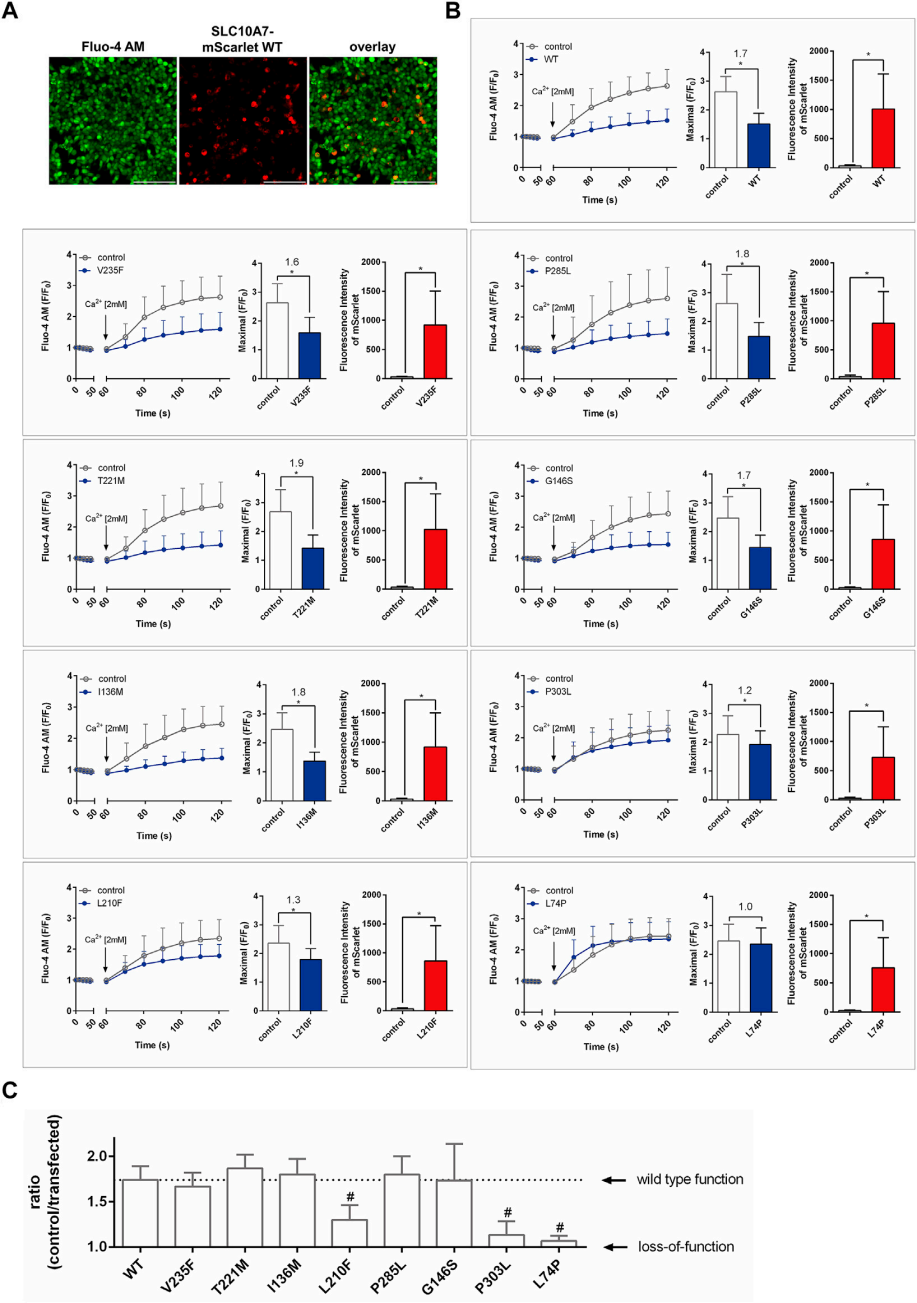
Effects of SLC10A7-mScarlet wild-type (WT) and mutant constructs on Ca^2+^ influx into HEK293 cells. All constructs were transiently transfected into HEK293 cells. After transfection, cells were prepared for calcium imaging by pre-incubation in Fluo-4 AM and thapsigargin (TG), followed by the addition of extracellular Ca^2+^. Red (mScarlet) and green (Fluo-4) fluorescence signals were recorded every 10 s. **(A)** Fluo-4 fluorescence signals were analyzed separately, (I) in additional red fluorescent cells (considered SLC10A7-mScarlet-expressing cells) and (II) non-red fluorescent cells (untransfected controls). **(B)** The left bar graphs represent the maximal induced Ca^2+^ fluorescence (maximum mean fluorescence at any time point) in both cell types, and the right bar graphs indicate the fluorescence intensities of the SLC10A7-mScarlet fusion proteins. Statistical analysis was performed using Student’s t test. * Significantly different at *p* < 0.01. **(C)** Effects of the different SLC10A7/RCAS variants on cellular Ca^2+^ influx. Ratios (Ca^2+^ influx in non-transfected cells vs Ca^2+^ influx in SLC10A7/RCAS-expressing cells) indicate the effect of the expressed SLC10A7/RCAS protein on the Ca^2+^ influx. Variants with ratios near the value of 1.0 are considered as loss-of-function variants. Statistical analysis was performed using one-way ANOVA. # Significantly different from WT at *p* < 0.01. Data represent means ± SD of approximately 230–256 individual cells from three independent experiments (*n* = 3). Scale bars: 214 µm.

Finally, all SLC10A7-mScarlet constructs were transiently transfected into HEK293 cells and used to measure Ca^2+^ influx in cells preloaded with Fluo-4 AM and pretreated with TG. Extracellular Ca^2+^ was added at a concentration of 2 mM, and red (mScarlet) and green (Fluo-4) fluorescence was recorded every 10 s for 1 min. As reported before, overexpression of the SLC10A7-mScarlet WT construct significantly limited Ca^2+^ influx, with a ratio of 1.7 (Karakus et al., 2020). Red fluorescence was used to discriminate between SLC10A7-expressing and non-expressing cells and to compare the expression of the respective mScarlet-tagged protein (Figure 4A). Apart from the WT SLC10A7/RCAS protein, the SLC10A7 variants previously described to be associated with human pathologies, namely, L74P and P303L, were functionally analyzed in HEK293 cells. Both constructs revealed expression of the red fluorescent SLC10A7-mScarlet protein at levels comparable to those of the WT, as indicated by red bars. However, both variants significantly lost their effect on Ca^2+^ influx. In the case of the L74P variant, the effect on Ca^2+^ influx was completely abrogated, which indicates a complete loss of function, as expected. The P303L variant had only a moderate effect on Ca^2+^ influx (Figure 4B) and compared to the WT this effect was significantly reduced (Figure 4C). The same was true for the novel variant L210F, which maintained less than 50% of the function of the WT SLC10A7 construct, which suggests a disease potential of the corresponding rs764016906 *SLC10A7* rare genetic variant, at least in a biallelic constellation with another disease-related SLC10A7 variant. In contrast, all other novel SLC10A7 variants, namely, V235F, T221M, I136M, P285L, and G146S, showed effects on Ca^2+^ influx (ratios 1.6–1.9) and protein expression comparable to those of the WT SLC10A7 protein (Figures 4B,C).

## Discussion

### SLC10A7/RCAS Overexpression Restricts the Ca^2+^ Influx After TG-Induced ER Depletion

The store-operated Ca^2+^ entry (SOCE) with its major components STIM and ORAI is a central mechanism in cellular Ca^2+^ signaling. Several regulatory factors of the STIM/ORAI complex have been described, including the CRAC channel regulator 2A (CRACR2A; Srikanth et al., 2010) and the SOCE-associated regulatory factor (SARAF; Palty et al., 2012). Recently, with SLC10A7/RCAS we identified an additional novel regulatory factor of the intracellular Ca^2+^ signaling (Karakus et al., 2020). Expression of the SLC10A7/RCAS protein was negatively correlated with SOCE and after transient transfection of SLC10A7/RCAS into HEK293 cells, we found co-localization with STIM, ORAI, and SERCA. Based on this, we hypothesize a role of SLC10A7/RCAS (I) in limiting the transport capacity of SERCA or (II) as a negative regulator of STIM and/or ORAI. These hypotheses about the molecular mechanism of SLC10A7/RCAS on cellular Ca^2+^ signaling have to be addressed in future studies. For the present study, we just used our previous finding that SLC10A7/RCAS overexpression restricts the Ca^2+^ influx after TG-induced ER depletion in HEK293 cells to functionally analyze the effect of several novel genetic variants of SLC10A7 and to predict their disease potential. In detail, the effects of two known disease-related SLC10A7 variants (L74P and P303L) and six novel potentially disease-related variants (V235F, T221M, I136M, L210F, P285L, and G146S) were analyzed. We first examined whether these variant proteins are sorted as the WT SLC10A7/RCAS protein, and then we determined their effects on SOCE.

### Subcellular Localization of SLC10A7/RCAS

The SLC10 carrier family currently consists of seven members, three of which are expressed in the plasma membrane, where they perform carrier-mediated uptake of bile acids (NTCP/SLC10A1 and ASBT/SLC10A2) and sulfated steroid hormones (SOAT/SLC10A6) (Geyer et al., 2006). All other SLC10 members (SLC10A3-A5 and SLC10A7) are dominantly expressed in intracellular structures, and their sorting behavior depends significantly on the cell line used for expression and the tag used for detection. Unfortunately, appropriate antibodies that would allow detection of native untagged SLC10 proteins are not available for most of these intracellularly expressed carriers, except for SLC10A4, which is localized in synaptic vesicles of cholinergic and monoaminergic neurons of the central and peripheral nervous systems in rats (Geyer et al., 2008; Burger et al., 2011). Therefore, sorting studies for the SLC10A7/RCAS protein still require the use of tagged protein constructs. In a previous study, we used a red fluorescent mScarlet-tagged SLC10A7 construct that had significant effects on SOCE after transient transfection into HEK293 cells (Karakus et al., 2020). The same construct was also used for the sorting and Ca^2+^ influx experiments in the present study. In subsequent co-localization studies of SLC10A7-mScarlet and STIM-GFP we found a high degree of overlay, which indicates that large part of the SLC10A7/RCAS protein is localized in the ER in close proximity to STIM. However, it should be mentioned that previous studies found different sorting of the SLC10A7/RCAS protein. Using different FLAG- and HA-tagged SLC10A7 constructs, Godoy et al. (2007) demonstrated that part of the SLC10A7 protein was co-localized with calnexin in the ER, whereas a fraction of the protein was also detected in the plasma membrane. Also, the SLC10A7/RCAS homolog CaRch1p of *Candida albicans* is at least partly localized in the plasma membrane (Alber et al., 2013). The latter localization was verified by the expression of a SLC10A7-FLAG construct in *Xenopus laevis* oocytes (Godoy et al., 2007). In another study, a V5-tagged SLC10A7 construct showed clear intracellular localization in U2OS cells (Bijsmans et al., 2012). Also, Arshikov et al. (2018) used a V5-tagged SLC10A7 construct to localize the SLC10A7 protein and found expression in the Golgi compartment in HeLa cells. They also demonstrated a Golgi localization of the SLC10A7/RCAS protein in human fibroblasts after lentiviral transfection of a V5-SLC10A7 construct (Ashikov et al., 2018). Based on this, the exact subcellular localization of the SLC10A7/RCAS protein requires further investigation with appropriate antibodies that allow sorting studies with untagged native proteins in different cell lines and under different experimental conditions.

In the present study, great effort was made to localize the SLC10A7-mScarlet construct that previously showed intact function as a regulator of cellular Ca^2+^ influx. We examined the co-localization of mScarlet-tagged SLC10A7/RCAS and GFP-tagged organelle markers in HEK293 cells and found the highest degree of co-localization with the ER marker. ER expression of SLC10A7-mScarlet was further verified by co-localization studies with STIM-GFP, which is typically located in the ER. However, it has to be mentioned that the degree of ER localization was slightly higher for STIM and SERCA compared to RCAS, which indicates that at least part of the SLC10A7/RCAS protein might also be sorted to the Golgi and plasma membrane. As a dynamic sorting regulation is known for STIM, all sorting studies were additionally performed after ER Ca^2+^ depletion by TG treatment. As expected, after treatment with TG STIM lost some of its co-localization with SERCA but increased its co-localization with ORAI, which reflects quite well the physiological regulation of STIM sorting under ER Ca^2+^ depletion (Gwack et al., 2007; Manjarres et al., 2010). This indicates that we used an appropriate cellular system to analyze SOCE. Unfortunately, we were not able to additionally analyze the co-localization of SLC10A7-mScarlet with SERCA-GFP, because despite intense effort this construct could not be created. Based on these sorting and co-localization experiments we propose SLC10A7/RCAS as a direct regulatory factor for STIM and SERCA, but most likely not for ORAI. As a potential regulatory mechanism, RCAS in the physiological state of the cell might limit STIM interaction with ORAI. An additional hypothesis is that SLC10A7/RCAS might negatively regulate Ca^2+^ sequestration via SERCA.

### Effects of the Novel SLC10A7/RCAS Variants on Sorting and Ca^2+^ Influx

The major aim of the present study was to functionally test six novel SLC10A7 variants as well as one disease-related variant (P303L) in a cellular system. For these experiments, we included the disease-related loss-of-function variant L74P as a control. We first investigated whether these variants are sorted identically as the WT protein and whether they show a similar degree of co-localization with STIM. It is interesting that the two disease-related variants, namely, L74P and P303L, showed a significantly higher degree of co-localization with STIM compared to the WT. This might indicate an effect of the mutation on normal sorting behavior that might also contribute to defective function of these SLC10A7/RCAS variant proteins (Dubail et al., 2018; Laugel-Haushalter et al., 2019). In contrast, all other novel variants showed sorting behavior comparable to that of the WT. Next, we analyzed the functional role of all variants in intracellular Ca^2+^ homeostasis. For this purpose, we tagged the corresponding SLC10A7 variants with the red fluorescent protein mScarlet and transiently transfected them into HEK293 cells. HEK293 cells inherently show very low SLC10A7 expression (according to the protein atlas, www.proteinatlas.org) and therefore are an appropriate model for transient overexpression of the respective variant SLC10A7/RCAS proteins. Ca^2+^ flux measurements were performed in cell culture plates with transiently transfected cells, where cells with SLC10A7-mScarlet-derived red fluorescence were classified as transfected and cells without red fluorescence were classified as untransfected controls. As previously described by Karakus et al. (2020), RCAS overexpression significantly reduced Ca^2+^ influx in cells pretreated with TG after the addition of 2 mM Ca^2+^ to the extracellular medium. It is interesting that for five of the novel SLC10A7 variants (V235F, T221M, I136M, P285L, and G146S) no significant effect on Ca^2+^ influx could be detected compared to the WT, which indicates that contrary to the bioinformatics prediction the function of these SLC10A7/RCAS variant proteins was not affected. In contrast, the disease-related variant P303L (Laugel-Haushalter et al., 2019) that had not been functionally analyzed thus far as well as the novel L210F variant showed significantly reduced activity but maintained a moderate inhibitory effect on Ca^2+^ influx. Based on this finding, these two variants can be classified as variants with reduced function. In the case of P303L, this might explain the milder disease phenotype compared to other loss-of-function variants; in the case of L210F, a disease phenotype might be predicted, at least in a biallelic setting. The L74P variant that was included as a control proved again its loss-of-function phenotype as described previously (Karakus et al., 2020).

### Disease Phenotype of Patients With *SLC10A7* Mutation

In addition to the already established disease-related *SLC10A7* variants (Ashikov et al., 2018; Dubail et al., 2018; Laugel-Haushalter et al., 2019), the occurrence of L210F might be considered in patients with skeletal dysplasia or amelogenesis imperfecta. In addition, other nonspecific symptoms might be considered as described for patients with *SLC10A7* mutation. For example, Dubail et al. (2018) described the L74P mutation in a young patient of Turkish origin. The child showed several symptoms of skeletal dysplasia with multiple dislocations, amelogenesis imperfecta, and facial abnormalities. Furthermore, a delay in speech development was observed in a follow-up examination. Similar clinical features were described for patients with other *SLC10A7* mutations (G130R, Q172*, exon Δ9, exon Δ10, and exon Δ9 + 10). Interestingly, Laugel-Haushalter et al. (2019) described a milder phenotype in a patient with the P303L variant that could be more comparable to the L210F variant.

In conclusion, the occurrence of variants in the *SLC10A7* gene should be considered in patients with skeletal dysplasia and amelogenesis imperfecta. In addition to the already established variants, the present study identifies another potential disease-related SLC10A7/RCAS variant, namely, L210F, which seems to be most frequent in South Asian populations.

## Data Availability

The original contributions presented in the study are included in the article/Supplementary Material, further inquiries can be directed to the corresponding author.

## Glossary

2D: two-dimensional
3D: three-dimensional
ASBT: apical sodium-dependent bile acid transporter
CRAC: calcium release activated channel
CRACR2A: CRAC channel regulator 2A
E.coli: Escherichia coli
ER: endoplasmic reticulum
GFP: green fluorescent protein
gnomAD: Genome Aggregation Database
HEK293 cells: GripTite 293 MSR cells
IP_3_: inositol 1,4,5-trisphosphate
NTCP: sodium/taurocholate cotransporting polypeptide
PBS: phosphate-buffered saline
PCC: Pearson’s correlation coefficient
PCR: polymerase chain reaction
PDB: Protein Data Bank
PFA: paraformaldehyde
PolyPhen: Polymorphism Phenotyping
RCAS: negative regulator of intracellular calcium signaling
SARAF: SOCE-associated regulatory factor
SERCA: sarco/endoplasmic reticulum calcium-ATPase
SIFT: Sorting Intolerant From Tolerant
SLC10A7: solute carrier family 10 member 7
SNP: single nucleotide polymorphism
SOAT: sodium-dependent organic anion transporter
SOCE: store-operated calcium entry
STIM: stromal interaction molecule
TG: thapsigargin
TMD: transmembrane domain
WT: wild-type
